# A combination of artemisinin, moxidectin, and doxorubicin drugs can selectively and efficiently induce apoptosis in acute lymphoblastic and chronic myeloid leukemia cells in vitro and ex vivo

**DOI:** 10.1007/s12032-026-03320-8

**Published:** 2026-07-24

**Authors:** Viviana Soto-Mercado, Miguel Mendivil-Perez, Marlene Jimenez-Del-Rio, Carlos Velez-Pardo

**Affiliations:** 1 Grupo de Neurociencias de Antioquia, Instituto de Investigaciones Médicas, Facultad de Medicina, Universidad de Antioquia (UdeA), Calle 62 No. 52-59, Torre 1, Laboratorios 411-412, Medellin, Colombia; 2 Grupo de Neurociencias de Antioquia, Departamento Formación Básica Profesional, Facultad de Enfermería, Universidad de Antioquia (UdeA), Calle 62 No. 52-59, Torre 1, Laboratorios 411-412, Medellín, Colombia

**Keywords:** Acute leukemia, Apoptosis, Caspase-3, Antimalarial, Antibiotic, Anthelmintic, Drug

## Abstract

Acute lymphoblastic (ALL) and chronic myeloid (CML) leukemias are blood cancers that often resist traditional chemotherapy and other treatments. This is likely due to their ability to evade apoptosis. Therefore, inducing apoptosis in leukemia cells using innovative drug combinations may be the most effective therapeutic approach. Methods for multidrug combinations involving three or more drugs are scarce and much more complex to analyze. To address this issue, we propose an effective concentration 50 (EC_50_)-based, three-step method. The first step determines the lowest EC_50_ for each drug (e.g., artemisinin, chloroquine, primaquine, mefloquine, ivermectin, moxidectin, doxorubicin, and minocycline) by analyzing four cell endpoints (e.g., cell cycle, sub-G1, mitochondrial membrane potential (ΔΨ_m_), autophagy (lysosomes), and cleaved caspase 3 (CC3)) on K562 cells. Step two involves establishing the deleterious effect of the EC_50_-based drug combination at concentrations of single drugs at 1-, ½-, and ¼-EC_50_, respectively, on K562 leukemia cells. Step three involves using the optimal combined drugs to evaluate the same cellular endpoints in other non-leukemic and leukemic cells. We found that the combination of AM (1 µM), MD (10 µM), and DR (1.5 µM), i.e., at ½ EC_50_, induced cell cycle arrest in the S (25% ± 13, *N* = 4) and G2/M (55% ± 18, *N* = 4) phases, a drastic loss of ΔΨ_m_ (81% ± 6, *N* = 4), high lysosome accumulation (82% ± 10, *N* = 4), and CC3 (83% ± 13, *N* = 4), as evidence of apoptosis in K562 and Jurkat cells and ex vivo ALL and CML cells. The combined drugs were innocuous to peripheral blood lymphocytes (PBLs) (S phase = 40%; G2/M = 26%; ΔΨ_m_ = 4%; lysosomes = 3%; CC3 = 4%; *n* = 3). Our approach to combining drugs has the potential to provide a new pharmacological treatment for leukemias.

**Supplementary Information:**

The online version contains supplementary material available at https://doi.org/10.1007/s12032-026-03320-8.

## Introduction

Acute lymphoblastic leukemia (ALL) and chronic myeloid leukemia (CML) are lymphoproliferative and myeloproliferative neoplasms, respectively, of precursor B (pro-B) and T (pro-T) cells [1] and abnormal myeloid cells that have fused the Abelson murine leukemia (ABL1) gene on chromosome 9 with the breakpoint cluster region (BCR) gene on chromosome 22 [2]. Despite the use of intensive classical chemotherapy and other available targeted therapies, approximately 15%−20% of children with pro-B or pro-T ALL relapse [3–6]. Similarly, 10%−15% of CML patients with the BCR-ABL1 fusion gene (i.e., Philadelphia-positive; [7]) do not respond to tyrosine kinase inhibitor (TKI) treatment [8, 9]. One possible explanation for this resistance is that both ALL and CML have developed effective mechanisms to evade apoptosis [10], such as genetic and metabolic mechanisms of resistance to cytostatic agents [11] or BCR-ABL1-dependent and -independent mechanisms [12, 13]. Therefore, inducing apoptosis in leukemia cells using innovative drug combination techniques may be an effective therapeutic approach. More research is necessary to determine the most effective drug combinations.

Drug repurposing, also referred to as drug repositioning, reprofiling, or re-tasking, is a strategy that identifies new uses for existing drugs, including those that are approved or in development, for conditions that are beyond their original intended purpose [14, 15]. Repurposing drugs offers several advantages over traditional drug development, including reduced development, time and cost, established pharmacological and toxicity profiles, and increased success rates. Additionally, repurposed drugs can extend commercial viability, open new markets, benefit from regulatory incentives and expedited approval pathways, and speed up drug discovery [16, 17]. In line with this, antimalarial [18] and anthelmintic agents [19, 20] have been proposed as repurposed drugs as potential treatment for leukemia. For instance, artemisinin (AM [21, 22]), chloroquine (CQ [23]), primaquine (PQ [24]), mefloquine (MQ [25]), ivermectin (IM [26, 27]), and moxidectin (MD [19]) have been found as efficient agents against leukemia.

Mechanistically, antimalarial and anthelmintic agents exhibit multiple antileukemic mechanisms of action, including cell cycle arrest, induction of apoptosis via ROS-dependent and -independent mechanisms, alteration of the autophagy lysosomal pathway (ALP), and mitochondrial damage, among others (Table 1). These mechanisms support the idea that targeting cell organelles (e.g., mitochondria and lysosomes) or generation of oxidative stress (OS) simultaneously could enhance the cell death response. It is therefore not surprising that AM combined with sorafenib (a kinase inhibitor) and venetoclax -a selective BCL2 inhibitor [28], CQ combined with metformin [29], or IM with adriamycin [30] have proven to be effective against leukemia. Thus, combining drugs is one of the most advantageous therapeutic strategies against leukemia (e.g. [31]),. Since combination drug therapy improves leukemia treatment outcomes, we also selected the antibiotics doxorubicin (DR) and minocycline (MC), as they are known to exhibit antileukemic activity via complex prooxidant mechanisms [32, 33]. Our goal was to determine the most effective drug combination for treating leukemia cells in vitro and ex vivo.

**Table 1:** Drug name, Chemical structure, PubChem compound identification number, and mechanism of action of selected antimalarial, anthelmintic, and antibiotic drugs effective against leukemia cells

Drug	Chemical Estructure	PubChem CID	Mechanism of Action	Ref.
*Antimalarial*				
Artemisinin (AM)		68,827	Cell cycle arrest Apoptosis through ROS-dependent and independent mechanisms Lysosomal disruption	[34]
Chloroquine (CQ)		2719	Lysosomal disruption	[23, 35]
Primaquine (PQ)		4908	ROS-dependent mechanism NF-κB inhibition	[24]
Mefloquine (MQ)		4046	Lysosomal disruption	[25]
*Anthelmintic*				
Ivermectin (IM)		6,321,424	Disruption of the mitochondrial functions Oxidative stress Chloride-dependent membrane hyperpolarization Cell death	[26, 27]
Moxidectin (MD)		9,832,912	Increase intracellular chloride Mitochondrial outer membrane permeabilization (MOMP) Autophagy arrest	[19, 36]
*Antibiotics*				
Doxorubicin (DR)		31,703	Apoptosis through ROS-dependent mechanism DNA Alkylating agent	[32]
Minocycline (MC)		68,858,974	Apoptosis through ROS-dependent mechanism	[33]
*Chelator & Prooxidant*				
TPEN		5519	Apoptosis through ROS-dependent mechanism	[37]
*Metalloid*				
As_2_O_3_		14,888	Apoptosis through ROS-dependent mechanism	[38]

Combination drug therapy offers several benefits: (i) it increases the overall potency of treatment, producing synergistic or additive effects that enhance outcomes beyond what single drugs can achieve. This enhances treatment effectiveness; (ii) it reduces the development of drug resistance; (iii) it allows for lower doses of each drug, decreasing toxicity and adverse events without sacrificing effectiveness; (iv) it can address diverse targets; (v) it can lower treatment failure rates and long-term healthcare costs by improving efficacy and reducing resistance development [39, 40]. One aim of drug combination methods is to identify additive, synergistic, or antagonistic combinations using a single endpoint such as cellular viability [41]. Several methods are available for analyzing two-drug combinations (e.g. [42–44]),. However, the analysis of three-drug (e.g. [42, 45]), or multidrug (e.g. [45]), combinations, i.e., the analysis of high-dimensional drug combinations, is much more complex than the analysis of two-drug combinations [46]. Moreover, drug combination analysis is further complicated by the fact that the term synergy is still controversial in the pharmacological field [47–49]. As an alternative approach, we propose an effective concentration (EC_50_)-based, three-step method (Table 2). The EC_50_ is defined as the concentration of a drug that gives a half-maximal response (50% of the maximal response) of a biological pathway [50]. The first step determines the lowest EC_50_ for each drug (e.g., artemisinin, chloroquine, primaquine, mefloquine, ivermectin, moxidectin, doxorubicin, and minocycline) at 0, 1, 10, 100 µM analyzing four cell endpoints (e.g., cell cycle and sub-G1, mitochondrial membrane potential (ΔΨ_m_), autophagy (lysosomes), and cleaved caspase 3 (CC3) by flow cytometry on K562 cells, thereby avoiding established synergy or additivity models. Theoretically, at least 4 different drugs may be combined. For this purpose, statistical analysis (e.g., One-way ANOVA, post hoc multiple comparison, least significant difference) will established the 4 drugs with the lowest EC_50_. Step two involves establishing the deleterious effect of the EC_50_-based drug combination at concentrations of single drugs at 1-, ½-, and ¼-EC_50_, i.e., high (H), middle (M), and low (L) concentrations, respectively, on K562 leukemia cells. Step three involves using the optimal combined drugs to evaluate the same cellular endpoints in other non-leukemic (e.g., peripheral blood lymphocytes (PBL), used as a control), and leukemia cell lines (Jurkat cells, which are immortalized human T lymphocytes derived from T-cell leukemia). As a proof of concept, bone marrow-derived acute lymphoblastic (ALL) and chronic myeloid leukemia (CML) cells from chemoresistance in pediatric leukemia patients are included and treated with the optimal drug combination.

**Table 2:** Description of the EC_50_-based, three-steps method of drug combination toxicity in leukemia cells

	Outcome	Analysis
Step One: *Determination of the lowest EC*_50_* for single drugs*		
• Resolve the concentration-response curves of single drugs (antimalaria (4), anthelmintic (2), antibiotics (2) Table 1) at 0, 1, 10, 100 µM in K562 cells (4 endpoints) • Use of TPEN and As_2_SO_3_ as control (Table 1)	• a × b × c = 10 × 4 × 3 = **120** Concentration-response data points (Figs. 1, 2, 3 and 4) • Lowest (EC_50_)_1_ drug1 is determined for SubG1 phase; (EC_50_)_2_ drug2 for ΔΨ_m_; (EC_50_)_3_ drug3 for lysosomes; and (EC_50_)_4_ drug4 for CC3 (Table 3)	• Best-curve-fit analysis (e.g., linear, exponential, or logarithmic curve fitting) • Determination of EC_50_ for each drug (Table 3) • The lowest statistically significant EC_50_ for each endpoint is determined using One-Way ANOVA, Post Hoc Multiple Comparison, and Least Significant Difference (LSD) analyses
Step Two: *EC*_*50*_*-based drug combination deleterious effect on K562 leukemia cells*		
• Expose the K562 cells to 1 (high), ½ (middle), ¼ (low)-EC_50_ concentration of drug1, drug2, drug3, drug4.	• **None** • **High** (µM) combination: 1 drug1, 1 drug2, 1 drug3, 1 drug4. • **Middle** (µM) combination: ½ drug1, ½ drug2, ½ drug3, ½ drug4. • **Low** (µM) combination: ¼ drug1, ¼drug2, ¼ drug3, ¼ drug4. • d x e x f = 4 × 4 × 3 = **48** Combination-response data • **4** Representative Histograms (SubG1 phase, ΔΨ_m,_ lysosomes, CC3) (Fig. 5) • Highest toxic effect of drug combination on K562 cells = optimal drug combination	• Statistical significance of the highest effect of drug combination on K562 cells was determined using one-way or two-way analysis of variance (ANOVA), followed by a *Tukey post hoc comparison* to analyze differences among multiple experimental groups. Intergroup differences were considered significant only if the *p*-value was 0.05 (*), 0.01 (**), or 0.001 (***)
Step Three: *Deleterious effect of EC*_*50*_*-based drug combination on Jurkat cell line*,* and ALL & CML leukemia cells*		
• Expose the Jurkat cell lines, bone marrow-derived ALL (*N* = 1) and CML (*N* = 1) cells from chemoresistance in pediatric leukemia patients, and peripheral blood lymphocytes (PBL, as a control) to the optimal drug combination	• *Example*: Optimal drug middle combination, i.e., ½ drug1, ½ drug2, ½ drug3, ½ drug4. • g x h x i x j = 4 × 4 × 2 × 3 **= 96** data points • **16** Representative histograms (Fig. 6) • Mean ± S.D. for each one of the endpoints • Optimal drug combination postulated as potential use in large number of ex vivo ALL and CML cases [51, 52] and in vivo model of leukemia [53]	• Statistical significance of the effect of drug combination on cells was determined using one-way or two-way analysis of variance (ANOVA), followed by a *Tukey post hoc comparison* to analyze differences among multiple experimental groups. Intergroup differences were evaluated by *t-Student test* and statistically significant if the *p*-value was 0.05 (*), 0.01 (**), or 0.001 (***)

a = total number of drugs; b = number of cellular endpoints; c = three independent experiments; d = total number of (µM) combinations (= four drug combination plus none); e = number of cellular endpoints; f = three independent experiments; g = Total number of cells (primary cells + cell lines + control); h = number of cellular endpoints; i = untreated and treated cells; j = three independent experiments

Using the EC_50_-based method, we determined that the lowest apoptogenic EC_50_ values were for artemisinin (AM) at 2 µM, moxidectin (MD) at 20 µM, and doxorubicin (DR) at 3 µM. These three drugs were then combined at low (0.5, 5, and 0.75 µM), middle (1, 10, and 1.5 µM), and high (2, 20, and 3 µM) concentrations and tested on K562 cells. The middle combination of AM, MD, and DR (M, ½ EC50) was the most effective at inducing apoptosis in K562 and Jurkat leukemia cells, as well as ex vivo acute lymphoblastic leukemia (ALL) and chronic myeloid leukemia (CML) cells. This was evidenced by sub-G1 (S) phase cell cycle arrest (25% ± 13%; *N* = 4), G2/M phase cell cycle arrest (55% ± 18%; *N* = 4), loss of ΔΨ_m_ (81% ± 6%; *N* = 4), increased accumulation of lysosomes (82% ± 19%; *N* = 4), and increased cleaved caspase 3 (CC3, 83% ± 13%; *N* = 4). The M combination was innocuous to peripheral blood lymphocyte (PBL) cells (S phase = 40%; G2/M = 26%; ΔΨ_m_ = 4%; lysosomes = 3%; CC3 = 4%; *N* = 1). In conclusion, combined artemisinin, moxidectin, and doxorubicin can selectively and efficiently induce apoptosis in acute lymphoblastic and chronic myeloid leukemia cells in vitro and ex vivo. These data establish this drug combination as a potential approach to treating ALL and CML.

## Materials and methods

### Experiments with cell lines and primary leukemia samples

K562 cells (ATCC; Catalog No. CCL-243TM) and Jurkat T Leukemia Cell Culture, clone E6–1 (ATCC; Catalog No. TIB-152R) were used as myeloid and lymphoblastic leukemia cell models, respectively. All cells were grown at 37 °C in a humidified atmosphere of 5% CO_2_ in air, in RPMI medium (cat# 61870036, Gibco, New York, NY, USA) supplemented with 10% FBS 100 U/mL penicillin and 100 µg/mL streptomycin (MP Biomedicals, cat# 091674049, Santa Ana, CA, USA). Peripheral blood samples were collected by intravenous sampling and mononuclear (lymphocytes) cells were isolated by density gradient centrifugation using Ficoll-based solution (Ficoll-Paque) from 1 randomly selected control (non-leukemic patient, age 14 years old). Bone marrow samples were provided from a chemo-resistant T-ALL (Tissue Bank Code # 54460) and a CML patient (Tissue Bank Code #44186). T-ALL patients’ clinical characteristics were reported in Ref. [54]. A CML female patient (TBC#44186), age (7 years), presented a white blood cell counts (67,320), blasts (10%), karyotype (46XX; (t(9;22)(q34.1;q11.2), pcr p19+, complex karyotype), and immunophenotype (CD13+, CD33+, CD34+, CD117+, Myeloperoxidase (MPO)+). Briefly, patients were admitted at the Department of Hematology of the Hospital San Vicente Fundación, Medellin, Colombia. Written informed consent was obtained from all patients following the Declaration of Helsinki and the study was approved by the Medicine Faculty of the University of Antioquia and the Ethics Committee for Research Act # 04–2018 (Approval date: 9 February, 2018). For blast cells culture, BM mononuclear cells (stored at −20 °C for about 5 years) were thawed and cultured in MarrowMAX™ Bone Marrow Medium (cat. 12260014, Gibco, New York, NY, USA) as described previously [54]. Peripheral blood lymphocytes (PBL, fresh) were also cultured in MarrowMAX™ Bone Marrow Medium. Then after, floating cells were separated from attached cells, transferred to a new culture flask. Finally, both cell lines, PBL or BM cells were seeded at 1× 10^6^ cells/cm^2^ and incubated with 0, 1, 10 and 100 µM Artemisinin (Cat. HY-B0094; MedChemExpress, Monmouth Junction, NJ, USA), Chloroquine (Cat. HY-17589 A; MedChemExpress, Monmouth Junction, NJ, USA), Mefloquine (Cat. HY-17437 A; MedChemExpress, Monmouth Junction, NJ, USA), Primaquine (Cat. HY-12651 A; MedChemExpress, Monmouth Junction, NJ, USA), Ivermectin (Cat. HY-15310; MedChemExpress, Monmouth Junction, NJ, USA), Moxidectin (Cat. HY-B0777; MedChemExpress, Monmouth Junction, NJ, USA), Minocycline (Cat. M9511, Sigma-Aldrich, Saint Louis, MO, USA), TPEN (N, N,N′,N′-Tetrakis(2-pyridylmethyl)ethylenediamine; Cat. P4413 Sigma-Aldrich, Saint Louis, MO, USA) and doxorubicin (Cat. 44583, Sigma-Aldrich, Saint Louis, MO, USA) or with 0, 1, 3, 5 and 10 µM Arsenic trioxide (Cat. A1010, Sigma-Aldrich, Saint Louis, MO, USA) for 24 h at 37 °C in a humidified atmosphere of 5% CO_2_. All reagents were prepared following manufacturer’s recommendations.

### Determination of DNA fragmentation and cell cycle by Flow Cytometry

DNA fragmentation was determined by using a hypotonic solution of PI. Cells were left untreated or treated with the above-mentioned molecules alone or in combination for 24 h. After treatments, cells (1 × 10^5^) were washed with PBS (pH 7.2) and stored in 95% ethanol overnight at −20 °C. Then, cells were washed and incubated in 400 µL solution containing Propidium Iodide (PI; 50 µg/ml), RNase A (100 µg/mL), EDTA (50 mM), triton X-100 (0.2%) for 30 min at 37 °C. The cell suspension was analyzed for PI fluorescence by using a BD LSRFortessa II flow cytometer (BD Biosciences). Quantitative data and figures were obtained using FlowJo 7.6.2 Data Analysis Software (accessed in June 2025). Cells entering the sub-G1 phase were used as a marker of apoptosis (DNA fragmentation). For cell cycle analysis, the sub-G1 population was subtracted from the total acquired events, and the Dean Jett Fox analysis was applied (root mean square (RMS) < 10). The experiment was conducted three times, and 10, 000 events were acquired for analysis.

### Flow cytometry gating strategy for cell cycle analysis

Cell cycle distribution was determined by flow cytometry following ethanol fixation and DNA staining with PI DNA-intercalating dye. Initially, debris was excluded based on FSC-A versus SSC-A gating. To ensure accurate DNA content measurement, doublets were rigorously excluded. Doublets and aggregates were removed by gating on singlets using FSC-A versus FSC-H, selecting only singlet events. The gated singlet population was then analyzed by plotting DNA fluorescence intensity as a histogram, and cell cycle phases (G0/G1, S, and G2/M) were quantified using the cell cycle model from FlowJo software. This gating strategy (supplementary Fig. 1) minimized artifacts derived from aggregates and ensured reliable estimation of cell cycle phase distribution [55].

### Analysis of mitochondrial membrane potential by flow cytometry

Cells were treated as described above. Then, to test the ∆Ψm, we incubated cells (1 × 10^5^) for 20 min at 37 °C in the dark with active mitochondria-accumulating dye deep red MitoTracker^®^ compound (20 nM, final concentration, Cat. M46753, Thermo Fisher Scientific, Waltham, MA, USA). Cells were then analyzed by using a BD LSRFortessa II flow cytometer (BD Biosciences). The experiment was conducted three times, and 10,000 events were acquired for analysis. Quantitative data and figures were obtained using FlowJo 7.6.2 Data Analysis Software.

### Flow cytometry gating strategy for MitoTracker analysis

For the analysis of mitochondrial membrane potential, cells stained with MitoTracker were acquired by flow cytometry and analyzed using a sequential gating strategy according to [56]. First, cellular events were identified based on forward and side scatter parameters (FSC-A vs. SSC-A) to exclude debris. The final gated population (supplementary Fig. 2) was used to evaluate mitochondrial membrane potential, which was quantified as MitoTracker high fluorescence percentage and represented as histograms.

### Analysis CASPASE 3 by flow cytometry

Flow cytometry acquisition was used to determine the percentage CASP-3 positive cells. After treatments, cells (1 × 10^5^) were washed and simultaneously incubated for 20 min at 37 °C in the dark with CASP-3 detection reagent, FLICA™ (Thermo Fisher Scientific, cat # V35118, Waltham, MA, USA), according to manufacturer’s recommendation. Cells were then analyzed by using a BD LSRFortessa II flow cytometer (BD Biosciences). The experiment was conducted three times, and 10,000 events were acquired for analysis. Quantitative data and figures were obtained using FlowJo 7.6.2 Data Analysis Software.

### LysoTracker Labeling

The lysosomal-associated autophagy was carried out as described previously [57]. Briefly cells were incubated with the cell-permeable, non-fixable, green, fluorescent dye LysoTracker Green DND-26 (50 nM, cat #L7526, Thermo Fisher Scientific, Waltham, MA, USA) for 30 min at 37 °C. Cells were then washed, and LysoTracker fluorescence was determined by flow cytometry using a BD LSRFortessa II flow cytometer (BD Biosciences, Franklin Lakes, NJ, USA). The experiment was conducted 3 times, and 10,000 events were acquired for analysis. Quantitative data and figures were obtained using FlowJo 7.6.2 Data Analysis Software (BD Biosciences, Franklin Lakes, NJ, USA, accessed in November 2024). The LysoTracker Green fluorescence that was above or below normal was considered as autophagy related [58].

### Data Analysis

In this experimental design, primary cells (three biological replicates) or cell lines were isolated and cultured according to Refs. [59, 60]. The cell suspension was pipetted at a standardized cellular density of 1 × 10^6^ cells per cm² into different wells of a 24 well plate. Cells (i.e., the biological and observational units) [61] were randomized to wells by simple randomization (sampling without replacement method), and then, wells (i.e., the experimental units) were randomized to treatments using a similar method. Experiments were conducted in three independent experiments. The data from individual repetition yield a value of *n* = 1 for the experiment, and this was repeated on three occasions blind to the experimenter and/or flow cytometer analyst for a final value of *n* = 3 (technical replicates) [61]. Based on the assumptions that the experimental unit (i.e., the well) data comply with independence of observations, the dependent variable is normally distributed in each treatment group (*Shapiro–Wilk test*), and there is homogeneity of variances (*Levene’s test*). Statistical analyses were calculated with GraphPad Prism 5.0 software (https://www.graphpad.com/; accessed on February 14, 2025). Data were illustrated as the mean ± SD. The statistical significance was determined by one-way or two-way analysis of variance (ANOVA) followed by *Tukey’s post hoc comparison* to compare the differences between the multiple experimental groups.

To calculate the effective concentration 50 (EC_50_) of drugs (1, 10, 100 µM) on K562 cells, a concentration (µM)-(%) response curve was plotted. This curve reflected four endpoints: cell cycle (subG1 phase), mitochondrial membrane potential (ΔΨ_m_), autophagy (lysosomal) activity, and activation of cleaved caspase 3 (CC3). The mean % value obtained from each of the endpoints was the result of three independent experiments (*n* = 3). After performing a best-curve-fit analysis (e.g., linear, exponential, or logarithmic curve fitting), the EC_50_ was calculated using the output formula of the curve-fitting model. Based on these data, the lowest statistically significant EC_50_ was determined for each endpoint using One-Way ANOVA, Post Hoc Multiple Comparison, and Least Significant Difference analyses. Intragroup differences were only considered significant if the p-value was 0.05 (*), 0.01 (**), or 0.001 (***). In theory, statistical analysis may allow for the combination of at least four different drugs, each one targeting a single endpoint.

Then, K562 cells were exposed to a combination of at least three different drugs at high (H), middle (M), and low (L) concentrations (i.e., 1-, ½-, and ¼-EC₅₀ concentrations, respectively) in three independent experiments (*n* = 3) to assess the same four cellular endpoints. Data were illustrated as the mean ± SD. Statistical significance was determined using one-way or two-way analysis of variance (ANOVA), followed by a *Tukey post hoc comparison* to analyze differences among multiple experimental groups. Intergroup differences were considered significant only if the *p*-value was 0.05 (*), 0.01 (**), or 0.001 (***). The combination showing the highest statistically significant intergroup differences in each endpoint was selected as the optimal combination of drugs. Lastly, the Jurkat cell line, as well as ex vivo ALL and CML cells (*N* = 1 each), were exposed to the optimal drug combination. Statistical significance was determined by comparing untreated versus treated cells using a *Student’s t-test*. Results were considered statistically significant at *p* < 0.05 (*), *p* < 0.01 (**), or *p* < 0.001 (***).

## Results

### Antimalarial, anthelmintic, and antibiotic drugs cause significant S phase arrest of the cell cycle and an increase in the SubG1 population in K562 cells

**Fig. 1:**
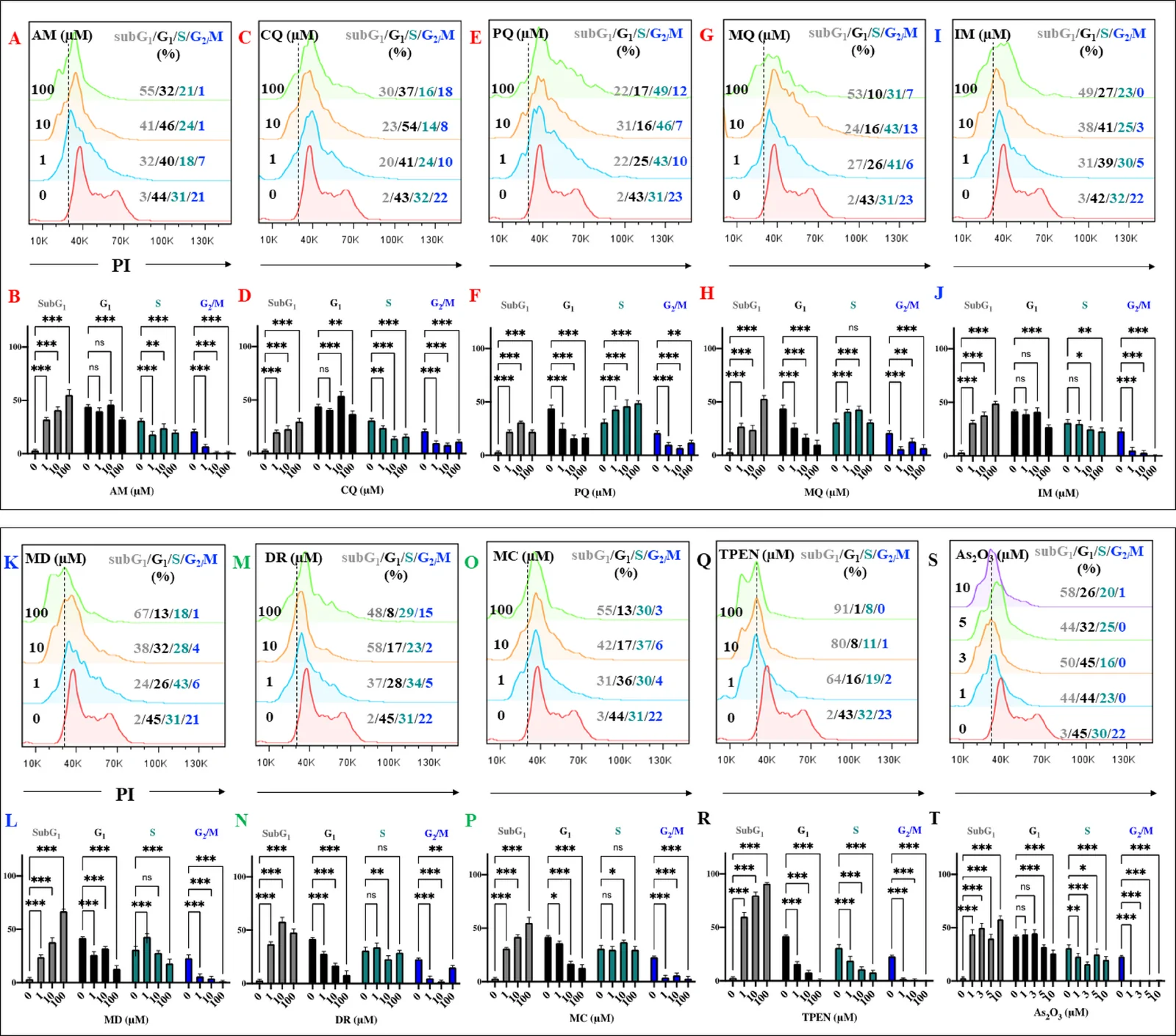
Antimalarial, anthelmintic, and antibiotic drugs affect the cell cycle and increase SubG1 phase in K562 cells. Representative histogram and quantitative data showing the SubG1, G1, S and G2/M positive populations in K562 cells treated with 0, 1, 10 and 100 µM artemisinin (AM; **A-B**), chloroquine (CQ; **C-D**), primaquine (PQ; **E-F**), mefloquine (MQ; **G-H**), ivermectin (IM; **I-J**), moxidectin (MD; **K-L**), doxorubicin (DR; **M-N**), minocycline (MC; **O-P**), N, N,N’,N’-Tetrakis(2-pyridylmethyl)ethylenediamine (TPEN; **Q-R**) and 0, 1, 3, 5 and 10 µM Arsenic trioxide (As_2_O_3_; **S-T**) evaluated with propidium iodide (PI) staining. Histograms represent 1 out of 3 independent experiments and quantitative numbers (0–100%) represent the PI positive mean percentage of three independent experiments (*n* = 3). Statistically significance at ^*^p < 0.05; ^**^p < 0.01; ^***^p < 0.001

Since leukemia cell division results in abnormal cell cycle progression and resistance to apoptosis [62], we first evaluated whether the tested drugs (listed in Table 1) altered the cell cycle and forced K562 leukemia cells to undergo apoptosis. As sub-G1 cell cycle arrest is a critical phase characterized by the lowest DNA content, indicative of DNA fragmentation and hypodiploidy (hallmarks of apoptosis), we used this phase to identify early apoptotic cells. Figure 1 shows that untreated K562 cells displayed a low percentage (~ 3%) of cells in the sub-G1, a high percentage in the G1 (~ 44%), S (~ 31%, indicating genomic DNA synthesis), and G2/M (~ 23%, indicating mitosis and proliferation) phases (Fig. 1A–T). Comparing the proportion of cells in the sub-G1 phase in untreated cells with the proportion induced by the maximum concentration of drugs (100 µM), it was found that the malarial agents AM, CQ, PQ, and MQ increased the Sub-G1 phase by + 1,733% (Fig. 1A and B), + 1,400% (Fig. 1C and D), + 1,000% (Fig. 1E and F) and + 2,550% (Fig. 1G and H), respectively, in K562 cells. Similarly, the anthelmintic agents IM and MD and the antibiotics DR and MC raised the Sub-G1 phase by + 1,533% (Fig. 1I and J), + 3,250% (Fig. 1K and L), + 2,300% (Fig. 1M and N), and + 1,733% (Fig. 1O and P), respectively. For comparative purposes, TPEN and As₂O₃ (used as controls) increased the Sub-G1 phase by + 4,450% (Fig. 1Q and R) and + 1833% (Fig. 1S and T), respectively. Furthermore, all drugs and control reagents caused systematic and significant dysregulation of the cell cycle in K562 cells, as demonstrated by G1, S, or G2/M phase arrest, as opposed to untreated cells (see Fig. 1A-T**).** It is worth mentioned that all drugs cause arrest of S phase and almost a complete disappearance of G2/M phases in K562 cells. Concentration-response analysis revealed that the lowest concentration at which the agents induced a half maximal effective concentration (EC_50_) Sub-G1 was 20 µM for the anthelmintic agent MD and 26 µM for the antibiotic DR (Table 3, Column A). However, while the EC_50_ was > 100 µM for CQ, PQ, MQ and IM, it was between 40 and 45 µM for AM and MC.

### Antimalarial drugs (primaquine and mefloquine), anthelmintic drugs (ivermectin and moxidectin), and antibiotics (doxorubicin and minocycline) induce dissipation of the mitochondrial membrane potential (ΔΨm) in K562 cells in a concentration-dependent manner

**Fig. 2:**
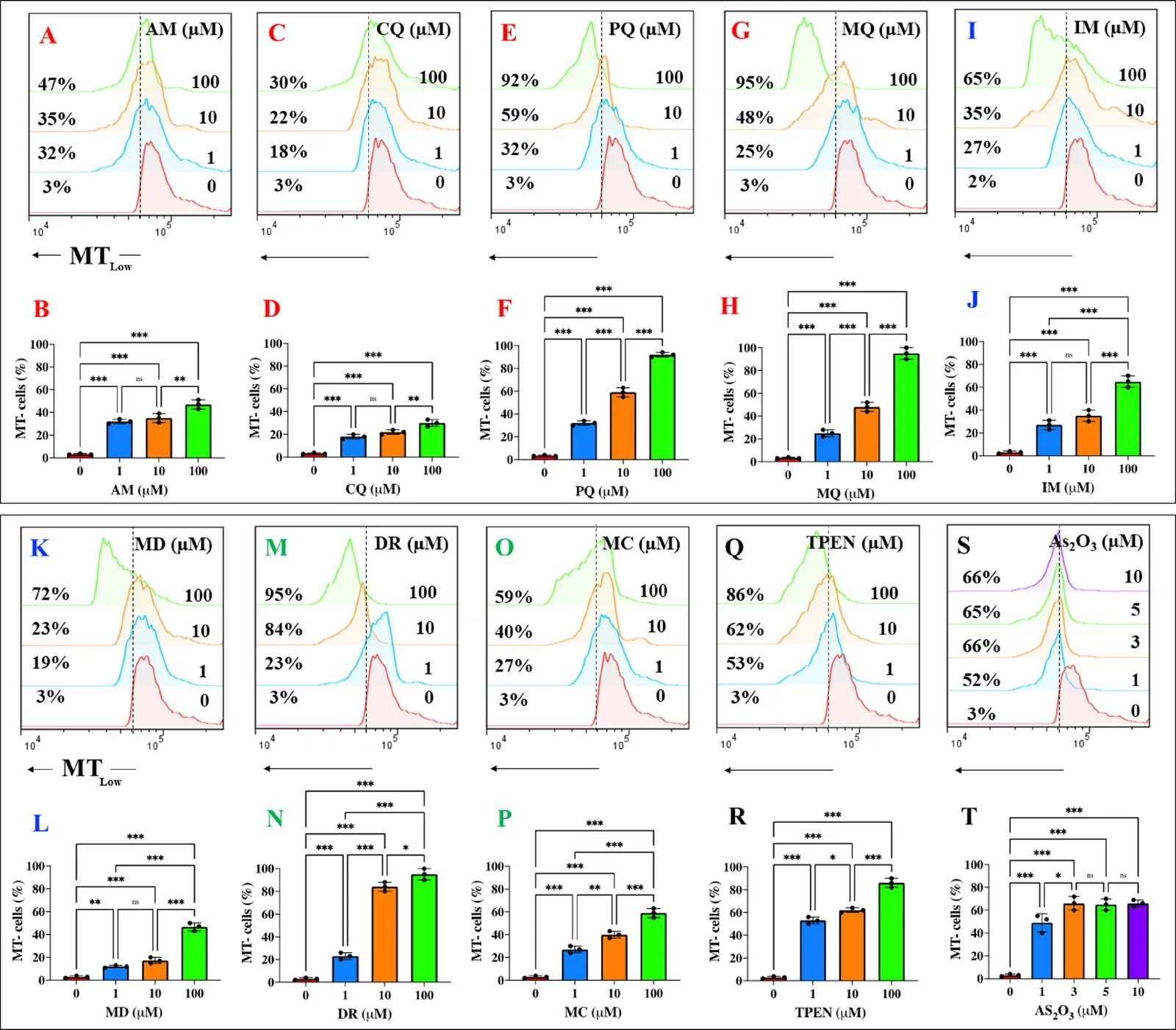
Antimalarial, antiparasitic, and antibiotic drugs induce the dissipation of mitochondrial membrane potential (ΔΨm) in K562 cells. Representative histogram and quantitative data showing the MitoTracker^®^ (MT) low populations in K562 cells treated with 0, 1, 10 and 100 µM artemisinin (AM; **A** and **B**), chloroquine (CQ; **C** and **D**), primaquine (PQ; **E** and **F**), mefloquine (MQ; **G** and **H**), ivermectin (IM; **I** and **J**), moxidectin (MD; **K** and **L**), doxorubicin (DR; **M** and **N)**, minocycline (MC; **O** and **P)**, TPEN (**Q** and **R**) and 0, 1, 3, 5 and 10 µM As_2_O_3_ (**S** and **T**). Histograms represent 1 out of 3 independent experiments and quantitative numbers (0–100%) represent the MT-low mean percentage of three independent experiments (black dots in bars). Statistically significance at ^*^p < 0.05; ^**^p < 0.01; ^***^p < 0.001

Mitochondria play a crucial role in a cell’s decision to die or survive [63]. Therefore, targeting mitochondria is a critical strategy for inducing apoptosis in acute leukemia [64]. We evaluated the effect of the drugs listed in Table 1 on ΔΨ_m_, which is generated by the electron transport chain and drives ATP synthesis via oxidative phosphorylation. Disruption therefore indicates mitochondrial dysfunction, which leads to apoptosis. As illustrated in Fig. 2, all drugs notably elevated the proportion of low MitoTracker^®^ green-fluorescent cells (MT-cells) relative to untreated K562 cells (Fig. 2A–P), suggesting dissipation of ΔΨ_m_ in a concentration-dependent manner, albeit with varying degrees of effectiveness. For example, at 100 µM, AM and CQ increased MT_low_ cells by up to + 1,467% (Fig. 2A and B) and + 900% (Fig. 2C and D), respectively, whereas PQ and MQ increased ΔΨm dissipation by + 2,966% (Fig. 2E and F) and + 3,066% (Fig. 2G and H). Similar to malarial drugs, the anthelmintic drugs IM and MD and the antibiotic agents DR and MC raised the percentage of MT_low_ cells to + 3150% (Fig. 2I and J), + 2,300% (Fig. 2K and L), + 3,066% (Fig. 2M and N), and + 1,866% (Fig. 2O and P), respectively, compared to untreated K562 cells. Similar behavior was observed with TPEN (+ 2,766, Fig. 2Q and R) and As₂O₃ (+ 2,100, Fig. 2S and T). Concentration-response analysis shows that the malarial drug PQ (EC_50_ = 4 µM) and the antibiotic agent DR (EC_50_ = 3 µM) were nearly as effective as the control reagents TPEN and As₂O₃, which have an EC_50_ = 1 µM (Table 3, column B).

**Table 3:** Effective concentration 50 (EC_50_) of antimalarial, anthelmintic, and antibiotic drugs on cell cycle (SubG1), loss of mitochondrial membrane potential (ΔΨm), lysosomes accumulation, and cleaved caspase 3 (CC3) in K562 cells

Column	A	Β	C	D
Endpoint EC_50_Drug	SubG_1_ µM	ΔΨm µM	Lysosomes µM	CC3 µM
*Antimalarial*				
Artemisinin (AM)	43**	> 100	> 100	2***
Chloroquine (CQ)	> 100	> 100	> 100	> 100
Primaquine (PQ)	> 100	4	> 100	11***
Mefloquine (MQ)	> 100	7	> 100	14***
*Anthelmintic*				
Ivermectin (IM)	> 100	25***	46	> 100
Moxidectin (MD)	**20**	28***	**19** ^***^	> 100
*Antibiotics*				
Doxorubicin (DR)	26	**3**	44	62
Minocycline (MC)	41**	32***	> 100	> 100
*Prooxidant*				
TPEN	0.1	1	> 100	1
*Metalloid*				
As_2_O_3_	5	1	85	7

The numbers (µM) represent the (rounded) value of concentration-response analysis and best-curve-fit analysis (e.g., linear, exponential, or logarithmic curve fitting). The name of drug and numbers in **bold** represent the selected drug with the lowest EC_50_ concentration for each endpoint and per drug category analyzed by One-way ANOVA, post hoc multiple comparison, least significant difference (LSD), α = 0.05; 0.01; 0.001. For practical comparison reasons, DR was considered as a control drug. Values > 100 µM were excluded from statistic intragroup comparisons. Statistically significant at ^*^p < 0.05; ^**^p < 0.01; ^***^ *p* < 0.001

### Antimalarial, anthelmintic, or antibiotic drugs impairs autophagy lysosomal pathway (ALP) in K562 cells

**Fig. 3:**
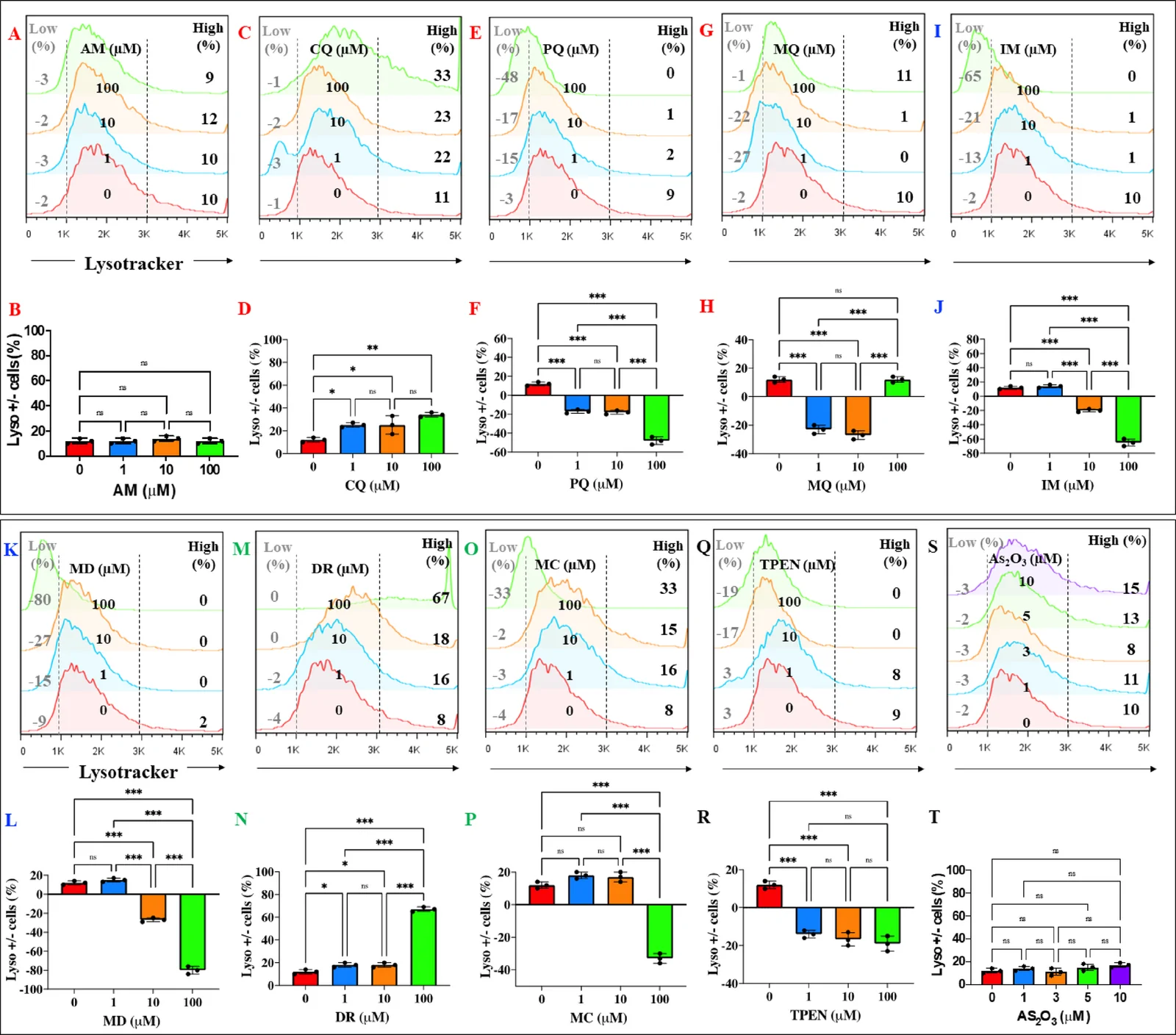
Antimalarial, antiparasitic, and antibiotic drugs increase accumulation of lysosomes in K562 cells. Representative histogram and quantitative data showing the LysoTracker positive and/or negative populations in K562 cells treated with 0, 1, 10 and 100 µM artemisinin (AM; **A** and **B**), chloroquine (CQ; **C** and **D**), primaquine (PQ; **E** and **F**), mefloquine (MQ; **G** and **H**), ivermectin (IM; **I **and** J**), moxidectin (MD; **K **and** L**), doxorubicin (DR; **M** and **N**), minocycline (MC; **O** and **P**), TPEN **(Q** and **R**) and 0, 1, 3, 5 and 10 µM As_2_O_3_ (**S** and **T**). Histograms represent 1 out of 3 independent experiments and quantitative numbers (0–100%) represent the mean percentage LysoTracker determination of three independent experiments (black dots in bars). Statistically significance at ^*^p < 0.05; ^**^p < 0.01; ^***^p < 0.001

Lysosomes are critical organelles for the ALP [65]. The use of LysoTracker probes (e.g., LysoTracker Green DND-26) has been employed to investigate the extent of autophagy by measuring their fluorescence via flow cytometry in K562 and Jurkat cells [58]. Figure 3(A–P) shows the effects of various drugs at increasing concentration on K562 cells. While AM (Fig. 3A and B) and As₂O₃ (Fig. 3S and T), all other drugs alter the ALP, as evidenced by either accumulation of lysosomes (CQ, Fig. 3C and D; DR, Fig. 3M and N), rapid clearance of lysosomes (PQ, Fig. 3E and F; IM, Fig. 3I and J; MD, Fig. 3K and L; MC, Fig. 3O and P; TPEN, Fig. 3Q and R), or a mix of both accumulation/clearance of lysosomes dependent on concentration (MQ, Fig. 3G and H). Concentration-response analysis revealed that the anthelmintic drug MD (EC_50_ = 19 µM) was the most effective at altering the ALP, followed by the anthelmintic drug IM (EC_50_ = 46 µM) and the antibiotic DR (EC_50_ = 44 µM) (Table 3, column C). The EC_50_ of the other drugs was greater than 100 µM.

### The activation of caspase-3 into cleaved caspase-3 (CC3) is induced by antimalarial, anthelmintic, and antibiotic drugs in K562 cells

**Fig. 4:**
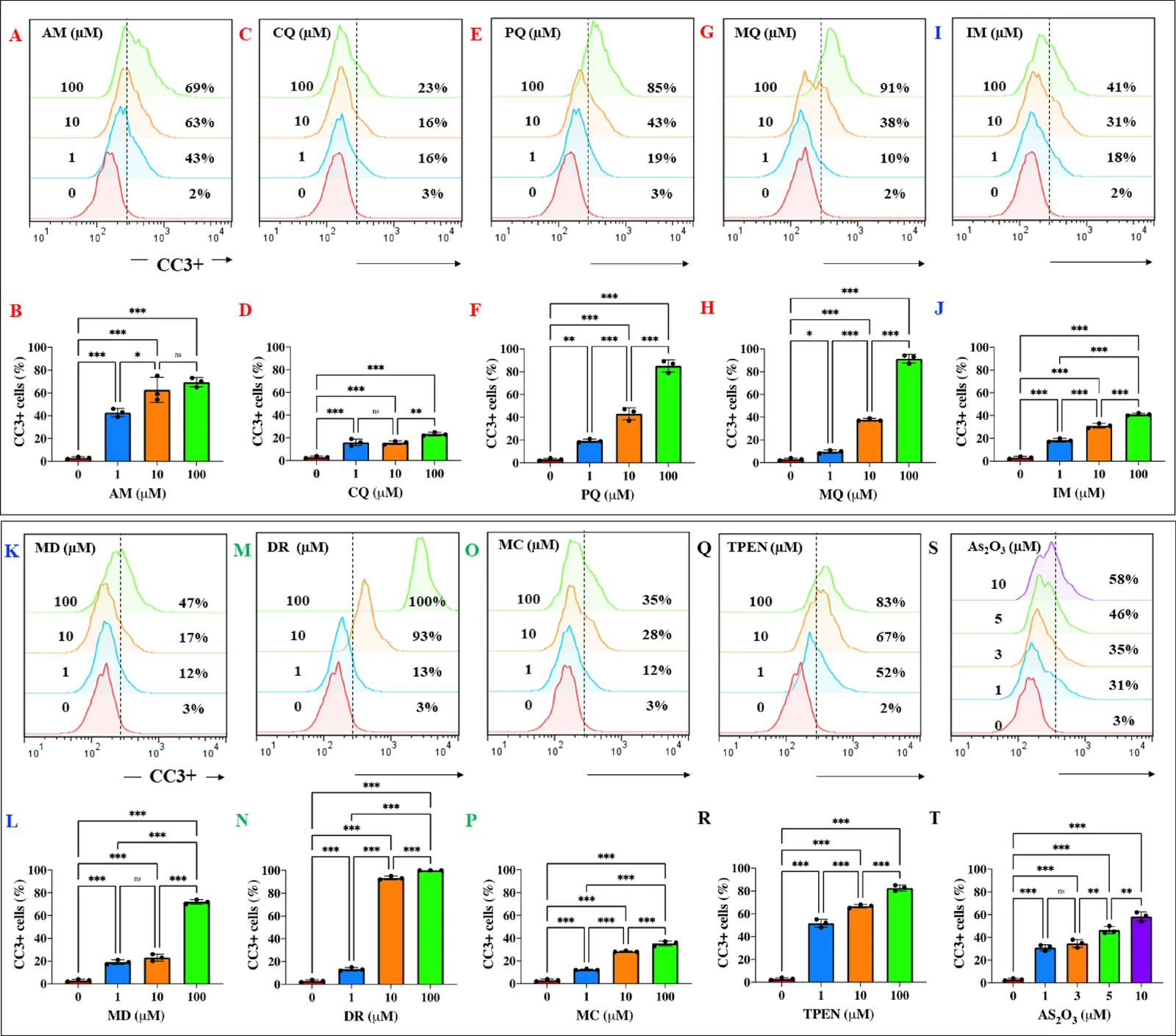
Antimalarial, antiparasitic, and antibiotic drugs induce the activation of the protease CASPASE 3 into cleaved caspase 3 (CC3) in K562 cells. Representative histogram and quantitative data showing the CC3 positive populations in K562 cells treated with 0, 1, 10 and 100 µM artemisinin (AM; **A** and **B**), chloroquine (CQ; **C** and **D**), primaquine (PQ; **E** and **F**), mefloquine (MQ; **G** and **H**), ivermectin (IM; **I** and **J**), moxidectin (MD; **K** and **L**), doxorubicin (DR; **M** and **N**), minocycline (MC; **O** and **P**), TPEN (**Q **and** R**) and 0, 1, 3, 5 and 10 µM As_2_O_3_ (**S** and **T**). Histograms represent 1 out of 3 independent experiments and quantitative numbers (0–100%) represent the mean percentage CC3 determination of three independent experiments (black dots in bars). Statistically significance at ^*^p < 0.05; ^**^p < 0.01; ^***^p < 0.001

Cleaved caspase 3 (CC3) is a reliable marker of apoptosis, commonly detected by flow cytometry [66]. As shown in Fig. 4, a low percentage (2–3%) of CC3-positive cells was observed in untreated K562 cells (Fig. 4A–T). Upon treatment with the increasing concentration of drugs listed in Table 1, the proportion of CC3-positive cells invariably increased form 1 up to 100 µM, albeit to varying degrees. Among the malarial drugs, at 100 µM, AM, CQ, PQ, and MQ increased CC3-positive cells by + 3,350% (Fig. 4A and B), + 667% (Fig. 4C and D), + 2,733% (Fig. 4E and F), and 4,450% (Fig. 4G and H), respectively, compared to untreated cells. Similarly, the anthelmintic drugs IM and MD and the antibiotic drugs DR and MC increased CC3-positive cells by + 1,950% (Fig. 4I and J), + 1,467% (Fig. 4K and L), + 3,233% (Fig. 4M and N), and + 1,067% (Fig. 4O and P), respectively. For reference, TPEN and As₂O₃ provoked CC3 cell activation by + 4,050% (Fig. 4Q and R) and + 1,833% (Fig. 4S and T), respectively. Overall, all compounds induced CC3 cells in a concentration-dependent manner (Fig. 4A–T). However, a concentration-response analysis showed that the malarial drug AM (EC_50_ = 2 µM) was as effective as the control reagent TPEN (EC_50_ = 1 µM) (Table 3, column D).

### A combination of artemisinin (AM), moxidectin (MD) and doxorubicin (DR) arrest the cell cycle at G2/M phase and induce accumulation of lysosomes while inducing dissipation of the mitochondrial membrane potential (ΔΨm) and activating the protease caspase-3 (CC3) in K562 cells

**Fig. 5:**
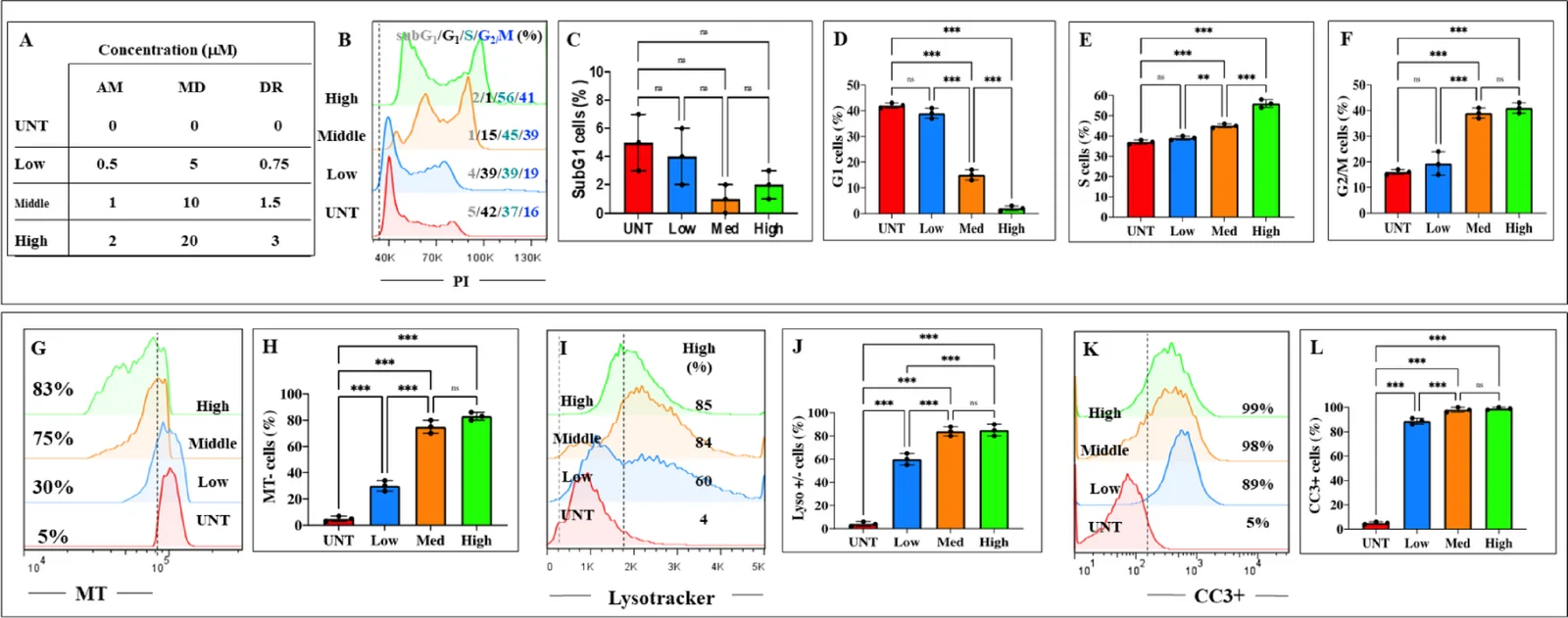
Combined artemisinin (AM), moxidectin (MD), and doxorubicin (DR) affect the cell cycle, increase SubG1, increase accumulation of lysosomes, induce the dissipation of mitochondrial membrane potential (ΔΨm) and activate the protease CASPASE-3 (CC3) in K562 cells. (**A**) Table showing the high (2 AM, 20 MD, 3 DR), middle (1 AM, 10 MD, 1.5 DR), and low (0.5 AM, 5 MD, 0.75 DR) concentrations (µM) of antimalarial, anthelmintic, and antibiotic drugs or untreated condition. Representative histogram and quantitative data showing the SubG1/G1/S/G2/M phases of the K562 cell cycle (**B**) in untreated (UNT) or treated with high, middle, and low concentrations of AM, MD, and DR. Quantification of the percentage of SuG1 (**C)**, G1 **(D)**, S **(E)**, G2/M **(F)** in untreated (UNT) or treated with different drug concentrations. Representative histogram showing MitoTracker^®^ green-fluorescent cells in untreated (UNT) or treated with different drug concentrations (**G**). Quantification of the percentage of MitoTracker^®^ green-fluorescent cells in untreated (UNT) or treated with different drug concentrations (**H**). Representative histogram showing LysoTracker low and/or high of lysosome green-fluorescent cells proportion in untreated (UNT) or treated with different drug concentrations **(I)**. Quantification of the percentage of LysoTracker low and/or high of lysosome green-fluorescent cells proportion in untreated (UNT) or treated different drug concentrations (**J).** Representative histogram showing the proportion of cleaved caspase 3 (CC3) positive cells in untreated (UNT) or treated with different drug concentrations (**K).** Quantification of the percentage of CC3 + cells in untreated (UNT) or treated different drug concentrations (**L).** Histograms represent 1 out of 3 independent experiments. The numbers represent the mean percentage of three independent experiments. Statistically significance at ^*^p < 0.05; ^**^p < 0.01; ^***^p < 0.0001

The above observations prompted us to investigate whether a combination of drugs could obliterate K562 cells. Based on the median EC_50_ of the drugs (Table 3, columns A–D), we selected those drugs that had the least EC_50_ effect (i.e., high potency) on the cell cycle, ΔΨ_m_, lysosomes, or CC3 induction. Given that some antimalarial, anthelmintic, or antibiotics had a mean EC_50_ > 40 or > 100 µM on those cell cycle variables **(**Table 3**)**, we selected MD, which efficiently increased the sub-G1 phase (column A) and significantly induced accumulation of lysosomes (column C); DR, which efficiently depolarized the ΔΨ_m_ (column B), and AM, which efficiently induced CC3 (column D). The selected antimalarial, anthelmintic, and antibiotic drugs were combined at low (L; 0.5 AM, 5 MD, and 0.75 DR), middle (M; 1 AM, 10 MD, and 1.5 DR), and high (H; 2 AM, 20 MD, and 3 DR) concentrations (µM; Fig. 5A). Untreated K562 cells exhibited a basal pattern of subG1/G1/S/G2M cell cycle phases (Fig. 5B), whereas treatment with L, M, or H combinations drastically affected the cell cycle. Notably, none of the combinations induced SubG1 (Fig. 5C**)**. While G1 decreased in step-wise from L to H combination (Fig. 5D**)**, the M and L arrested cell cycle at S **(**Fig. 5E**)** and G2/M **(**Fig. 5F**)** phases more efficiently than the L combo. MitoTracker fluorescent analysis revealed that the L, M, and H combinations induced a loss of ΔΨ_m_ by + 500%, + 1,400%, and + 1,560%, respectively, compared to untreated K562 cells (Fig. 5G). M and H induced significantly greater depolarization of the ΔΨm than the L combination (Fig. 5H). Similar to MitoTracker, the L, M, and H combinations increased the accumulation of lysosomes by + 1,400%, + 2,000%, and + 2,025%, respectively, compared to untreated K562 cells (Fig. 5I). Furthermore, M and H induced a significant increase in vesicular lysosomal accumulation to a greater extent than the L combination (Fig. 5J). Cleaved caspase 3 (CC3) detection showed that the L, M, or H combinations induced apoptosis by between + 1,680% and + 1,880% (Fig. 5K). The M and H combinations were equally effective at inducing CC3 in K562 cells (Fig. 5L). Overall, the M and H combinations efficiently dismiss K562 cells. As there was no significant statistical difference between the M and H treatments, the M combination was selected for further experiments.

### A combination of the antimalarial agent artemisinin (AM), the anthelmintic agent moxidectin (MD), and the antibacterial agent doxorubicin (DR) selectively and efficiently induce apoptosis in leukemia cells

**Fig. 6:**
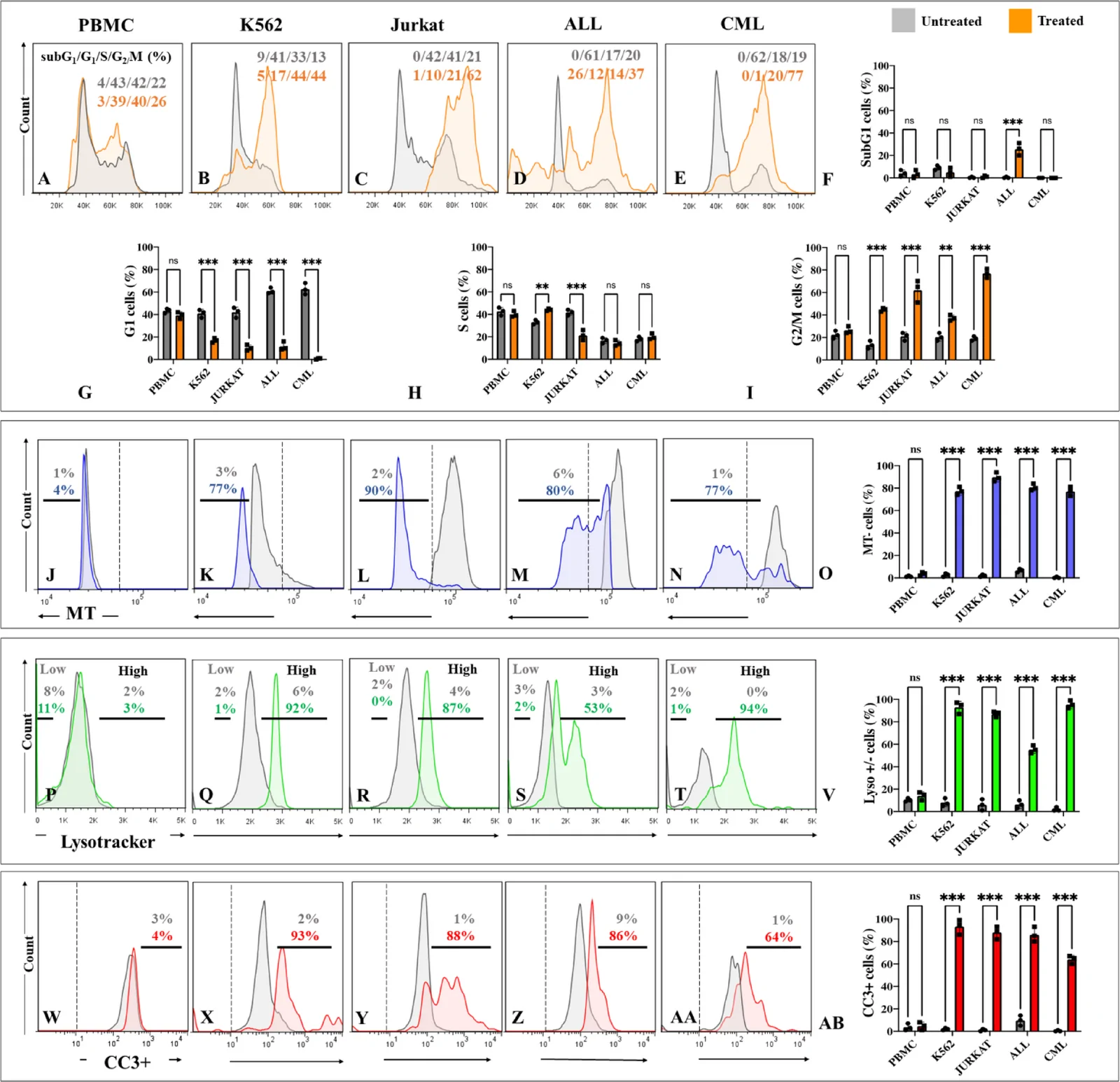
Effect of middle concentration of artemisinin (AM), moxidectin (MD), and doxorubicin (DR) on cell cycle progression, mitochondrial membrane potential (ΔΨ_m_) dissipation, activation of the protease CASPASE-3 (CC3), and lysosomal accumulation in human peripheral blood lymphocytes (PBLs), Jurkat and K562 cells, and bone marrow-derived acute lymphoblastic (ALL) and chronic myeloid Leukemia (CML) cells from chemo resistant pediatric leukemia patients. Representative histogram and quantitative data showing the SubG1, G1, S and G2/M positive populations (**A-I)**, MitoTracker (MT) low **(J-O)**, LysoTracker positive and/or negative **(P-V)**, the active protease CASPASE-3 (CC3**) (W-AB)** in PBL **(A**,** F-J**,** P**,** O**,** V**,** W**,** AB)**, K562 **(B**,** F-I**,** K**,** O**,** Q**,** V**,** X**,** AB)**, Jurkat **(C**,** F-I**,** L**,** O**,** R**,** V**,** Y**,** AB)**, ALL **(D**,** F-I**,** M**,** O**,** S**,** V**,** Z**,** AB)** and CML **(E**,** F-I**,** N**,** O**,** T**,**V**,** AA**,** AB)** cells untreated or treated with middle (AM, MD, and DR) concentrations of artemisinin (AM; 1 µM), moxidectin (MD; 10 µM) and doxorubicin (DR; 1.5 µM). Histograms represent 1 out of 3 independent experiments and quantitative numbers (0–100%) represent the mean percentage of three independent experiments (black dots in bars). Statistically significance at ^*^p < 0.05; ^**^p < 0.01; ^***^p < 0.001

To prove the concept, we used AM, MD and DR in (M) combination against peripheral blood mononuclear cells (PBMC), acute lymphoblastic leukemia (ALL) Jurkat cells, ex vivo ALL and chronic myeloid leukemia (CML) cells. K562 cells were included as a reference (**see** Fig. 5). Cell cycle analysis showed that PBMC exhibited a similar pattern in the cell cycle (SubG1/G1/S/G2/M phases) in both untreated and M combo-treated PBMC (Fig. 6A and F–I). However, except for ex vivo ALL cells (Fig. 6D and F), the M combination did not induce a statistically significant difference in the sub-G1 phase in the other leukemia cells compared to untreated cells (Fig. 6B–F). Nevertheless, the M combination induced significant cell arrest at the S and G2/M phases in K562 cells (Fig. 6B and G, and 6H), and cell arrest at G2/M phases in Jurkat cells (Fig. 6C and G), ex vivo ALL cells (Fig. 6D and G), and CML cells (Fig. 6E and G). MitoTracker^®^ fluorescent analysis revealed that PMBC was almost unperturbed by exposure to the M combo compared to untreated cells (Fig. 6J and O). By contrast, the M combo increased depolarization of ΔΨm by + 2,466% and + 4,400% in both K562 cells (Fig. 6K and O) and Jurkat cells (Fig. 6L and O), respectively, as well as by + 1,233% and + 7,600% in ex vivo ALL cells (Fig. 6M and O) and CML cells (Fig. 6N and O), respectively, compared to untreated cells. Analysis of LysoTracker^®^ green fluorescence dye and detection of cleaved caspase 3 (CC3) show that the M combo did not alter the vacuolar lysosomes (Fig. 6P) or caspase 3 activity (Fig. 6W) in PBMC compared to untreated cells (Figs. 6V andAB). However, upon exposure to the M combo, K562 (Fig. 6Q and X), Jurkat (Fig. 6R and Y), ALL (Fig. 6S and Z), and CML (Fig. 6T and AA) cells showed a dramatic accumulation of lysosomes of between 1,433% and 1,667% (Fig. 6V), as well as an increase in CC3-positive cells of between 4,550% and 8,700% (Fig. 6AB), compared to untreated cells.

## Discussion

This study demonstrates for the first time that, out of eight drugs (e.g., AM, CQ, PQ, MQ, IM, MD, DR, and MC), a combination of three drugs (1 µM AM, 10 µM MD, and 1.5 µM DR) efficiently induces apoptosis (> 80%) in leukemia cell lines (K562 and Jurkat), as well as in ex vivo ALL and CML cells. This is evidenced by S and G2/M phase cell cycle arrest, depolarization of the mitochondrial membrane potential (ΔΨ_m_), accumulation of lysosomes, and cleaved caspase 3 (CC3), which is indicative of apoptosis. Therefore, this combination represents a potential drug target for leukemia treatment. We reached this conclusion based on the following observations: First, we adopted a simple combination method consisting of three basic steps. The first step estimates the concentration-response curve for each drug and calculates the EC_50_ according to 4 endpoints: cell cycle arrest, loss of mitochondrial membrane potential, dysfunction of lysosome, and the activation of caspase 3 for each drug as quantal effect data (0%−100%). These four endpoints represent the main targetable biological hallmarks of cancer cells, i.e., sustained proliferative signaling, deregulated autophagy lysosomal pathway and metabolism, and resistance to cell death [67]. After statistical analysis, the selection of drugs with the lowest EC_50_ (i.e., the highest potency) to affect each cellular endpoint is established. Since the lowest EC_50_ values may be similar between drugs (e.g., MD and DR affect the cell cycle and autophagy, and PQ and MQ affect ΔΨ_m_ and CC3), the likelihood of a drug combination increasing is high. The investigator has, at least, two options: (i) Select three or more drugs with a low EC_50_ for a drug combination. (ii) Select the lowest EC_50_ at each endpoint with a maximum of four drugs for the drug combination. We selected the latter option. However, MD affected both the cell cycle (SubG1 phase) and autophagy (lysosomes). Together with DR and AM, MD made up a three-drug combination. Step two determines the deleterious effect of the EC_50_-based drug combination at concentrations at 1-, ½-, and ¼-EC_50_, respectively, on K562 leukemia cells. This step reduced the number of drug mixtures to three combinations (high, middle, and low). In contrast, other combination experiments require at least thirty mixtures of three drugs [46]. Step three involves using the optimal combined drugs (established in step two) to evaluate the same cellular endpoints in other non-leukemic and leukemic cells.

Second, the EC_50_-based method has advantages over other combination methods, such as the isobologram and Chou-Talalay methods, because it uses EC_50_ as the minimum drug potency. If mixed with other drugs at ½ or ¼ of EC concentration, it is reasoned that the combined drugs will have a varied range of cellular effects. This avoids the need for complex mathematical calculations, such as the combination index for more than three compounds [42, 43]. Third, since the definition of a synergistic interaction is still under debate [10], classifying the effect of combined drugs as synergistic, additive, or sub additive seems problematic. We propose defining the effect of combined drugs by the quantal effect: 0% for no observed adverse effect, 1%−25% for the lowest observed effect, 26%−50% for a low effect, 51%−75% for a moderate effect, and 76%−100% for a strong effect (in terms of apoptosis-induced cell death). Accordingly, the combination of AM, MD, and DR might be defined as having a strong adverse effect or as having maximal potency in inducing apoptosis in leukemia cells. Fourth, a major difficulty with the synergy methodology is that it is virtually impossible to detect synergy among mixtures of more than two drugs or in a multidrug mixture. The present method of analysis clarifies this uncertainty because the mechanism of action of each repurposed drug in the combination is known (see Table 1 and references). However, this interpretation must be regarded with caution. In theory, if each drug acts independently on a different cellular target, we could select four drugs to combine (e.g., one drug affects the cell cycle, another affects the mitochondria, another affects the lysosomes, and a fourth induces CC3). However, this may not be the case. For example, as mentioned above, MD has been shown to affect multiple targets (e.g., the cell cycle and the lysosome) with high potency, while PQ and MQ affect ΔΨ_m_ and CC3 with similar high potency. Fifth, as a typical concentration-response assessment, we established that all drugs impaired cell cycle, increased the SubG1, caused loss of ΔΨ_m_, increased lysosome accumulation, and activated CC3 at the highest tested concentration (100 µM) in K562 cells. These observations suggest that leukemia cells respond to noxious stimuli by activating specific death pathways. Finally, since the present combination analysis relies on four cellular endpoints that define cell death by apoptosis (i.e., cell cycle, mitochondria, autophagy, and CC3), this method seems more robust for determining the most effective combination for treating leukemia cells in vitro and ex vivo than methods that use only one endpoint, e.g., 3-(4,5-dimethylthiazol-2-yl)−2,5-diphenyl-2 H-tetrazolium bromide (MTT) assay to reflect cell viability [68], 7-Aminoactinomycin D **(**7-AAD) staining to reflect cell membrane injury [46], or xCELLigence RTCA DP system to reflect cell viability in real-time [69], in single-dose response studies, two-drug or multi-drug combination studies. Taken together, our findings suggest that at least three drugs can be selected from each endpoint based on their lowest EC_50_ values and known mechanisms of action. This approach targets vital intracellular organelles or functions, such as cell cycle, mitochondria and the autophagy-lysosomal system, in a cost-effective manner (e.g., flow cytometry analysis).

What is the mechanism (or mechanisms) by which the three drugs efficiently kill leukemia cells? Although not directly tested in our experimental settings, our observations suggest that DR, AM, and MD induce apoptosis in leukemia cells through different mechanisms. These mechanisms may include DR-induced oxidative stress (OS) [70], AM-induced OS and lysosomal dysfunction [71], and MD-induced autophagy arrest reflected by increased lysosomal accumulation [72]. Further investigation is therefore required to fully understand the toxicity of the combined drugs. Nonetheless, DR is currently thought to act as a DNA alkylating agent, affecting primarily the S phase of the cell cycle by interfering with DNA replication and causing DNA damage. This damage can lead to cell cycle arrest mediated by p53, particularly in the S and G2/M phases, and ultimately induce apoptosis [73]. It is worth mentioning that K562 cells lack p53 protein expression [74, 75]. However, p73 -a p53 family member- fully replaces p53 [76, 77]. Therefore, DR-induced apoptosis in leukemia cells is warranted by the expression of either the p53- or p73-expressing protein (e.g., Jurkat or K562 cells, respectively). In addition, DR disrupts topoisomerase II-mediated DNA repair [78] and inhibits transcription elongation [79]. In agreement with those observations, we found that at 1.5 µM of combined DR disrupts the cell cycle by arresting the S and G2/M phases in leukemia cells (e.g., Jurkat cells [80]; this study). DR may also act as a prooxidant molecule [81]. Indeed, DR oxidation into semiquinone, which then converts back to doxorubicin, may contributes to ROS generation, primarily hydrogen peroxide (H₂O₂). This compound acts as a second messenger [82] that triggers a cascade of molecular events leading to apoptosis in leukemia cells [32]. Overall, DR induces apoptosis in leukemia cells through mitochondria-dependent and -independent mechanisms, as evidenced by cell cycle arrest (S and G2/M phases), loss of ΔΨ_m_, and a high percentage of CC3-positive cells. We also report that DR induces a modest aberrant accumulation of lysosomes. MD induces autophagy disruption [36] and depolarization of the ΔΨ_m_ (EC_50_ = 28 µM) in leukemia cells [19]. This creates a mitophagy imbalance, which is a vital lysosomal degradative pathway [83]. The imbalance further aggravates cell death by apoptosis, as reflected by an increasing proportion of CC3-positive leukemia cells. Of note, 1 µM of AM alone increased the proportion of SubG1 (32%, Fig. 1), loss of ΔΨ_m_ (32%, Fig. 2), and CC3 (43%, Fig. 4) markers. However, little alteration of lysosomes, even at higher concentrations (10–100 µM), was detected in K562 cells. These observations suggest that AM is a potent drug that induces apoptosis independently of autophagy. Interestingly, the combination of three drugs (1 µM AM, 10 µM MD, and 1.5 µM DR) induces cell cycle arrest in the S and G2/M phases. This combination also dramatically increases the percentage of cells with decreased ΔΨ_m_ (70%−90%), lysosome accumulation (53%−94%), and CC3 (64%−93%) compared to untreated cells (3%). Taken together, our findings suggest that the antileukemic drugs work co-jointly to eliminate 83% ± 13% (*N* = 4) of leukemia cells in vitro or ex vivo, as determined by the CC3 marker.

## Conclusion

We demonstrate here that a three-drug combination of antimalarial, anthelmintic, and antibiotic agents can effectively treat leukemia. Since leukemia cells involve complex intracellular networks, interventions must target multiple sites with multiple drugs. However, our findings suggest that weakening and disaggregating the apoptosis-resistant system by targeting mitochondria alone may be sufficient. Indeed, the loss of ΔΨ_m_, which is mainly caused by drug-induced ROS, induces either apoptosis (mitochondria-dependent mechanisms) or dysregulation of the autophagy-lysosomal pathway, as reflected by lysosomal accumulation (mitochondria-independent mechanisms). Overall, targeting mitochondria may be necessary and sufficient to destroy leukemia cells [84, 85]. Although the drug’s effect is concentration-dependent, calculating the EC_50_ reduced the number of concentrations of an individual drug that needed to be examined, thereby reducing the number of combinations. Furthermore, our approach minimized the high-dimensional experimental protocols. In this study, flow cytometry provided objective, quantitative, and statistically robust benefits [86]. Moreover, as an analytical tool for apoptosis, flow cytometry offers a means to calculate EC_50_ reproducibly, reliably, and comparably in a cost-effective manner by analyzing four typical cellular endpoints (i.e., cell cycle, ΔΨ_m_, lysosome, and CC3). Compared with other cell death assay methods [87], flow cytometry enables quantitative and qualitative analysis of signaling events with greater sensitivity and precision at the single-cell level [88], following a three-step protocol for detecting e.g., CC3-positive cells (e.g. [89]),. Since the pharmacokinetics (PK), toxicity, and therapeutic window of artemisinin, moxidectin, and doxorubicin are already known [90–92], future studies are warranted on the effects of sub-EC_50_ concentrations in an in vivo leukemia model (e.g. [53]),. Our findings suggest that discovering new antileukemic drug combinations is realizable. However, further studies are needed to confirm this assumption [93].

Despite these advantages, this work has some limitations. First, the small sample size of ex vivo leukemia cases (*N* = 2) is insufficient to draw definitive conclusions about the practicality of the EC_50_-based method. These samples were only used to prove the concept. Therefore, future studies should enlarge the number of ALL and CML cases to achieve statistical significance, as described in references [94, 95]. Second, although we demonstrated that the three-drug treatment caused an accumulation of lysosomes in leukemia cells using LysoTracker^®^ intensity alone, this method may not accurately reflect changes related to autophagy or autophagic flux. Thus, further studies involving the detection of additional autophagy markers, such as Beclin-2 and light chain 3-II (LC3-II) [96], are necessary to fully characterize the combined, drug-induced dysregulation of the autophagy-lysosomal pathway in leukemia cells [97]. Finally, although the EC_50_-based method is optimal for selecting the most effective three-drug combination (see Table 2**and** Table 3), other combinations are possible: primaquine (PQ) or mefloquine (MQ), moxidectin (MD), and doxorubicin (DR). Thus, these combinations could be tested on leukemia cell lines and in ex vivo leukemia cases.

## Supplementary Information

Below is the link to the electronic supplementary material.

Supplementary Material 1

## Data Availability

The data used to support the findings of this study are available from the corresponding author upon request.

## Sample availability

Not available.
