# Supplementary material for: A combination of artemisinin, moxidectin, and doxorubicin drugs can selectively and efficiently induce apoptosis in acute lymphoblastic and chronic myeloid leukemia cells in vitro and ex vivo
Source: Med Oncol. 2026 Jul 24;43(9):233. doi: 10.1007/s12032-026-03320-8 (PMC13400675; doi:10.1007/s12032-026-03320-8)
Supplement: Supplementary file 1 — Supplementary Material 1 [file 12032_2026_3320_MOESM1_ESM.docx]

Viviana Soto-Mercado^1^, Miguel Mendivil-Perez^2^, Marlene Jimenez-Del-Rio M^1,^ Carlos Velez-Pardo^1,*^. A combination of artemisinin, moxidectin, and doxorubicin drugs can selectively and efficiently induce apoptosis in acute lymphoblastic and chronic myeloid leukemia cells in vitro and ex vivo.

^1^Neuroscience Research Group, Medical Research Institute, Faculty of Medicine, University of Antioquia (UdeA), Calle 70 No. 52–21, and Calle 62 # 52–59, Building 1, Room 412; SIU Medellin, Colombia;

VS-M: viviana.soto@udea.edu.co;

CV-P: calberto.velez@udea.edu.co;

MJ-del-Rio: marlene.jimenez@udea.edu.co

^2^ Neuroscience Research Group, Faculty of Nurse, University of Antioquia (UdeA), Calle 70 No. 52–21, and Calle 62 # 52–59, Building 1, Room 412; SIU Medellin, Colombia;

MM-P: [miguel.mendivil@udea.edu.co](mailto:miguel.mendivil@udea.edu.co)

**Supplementary Material**

**
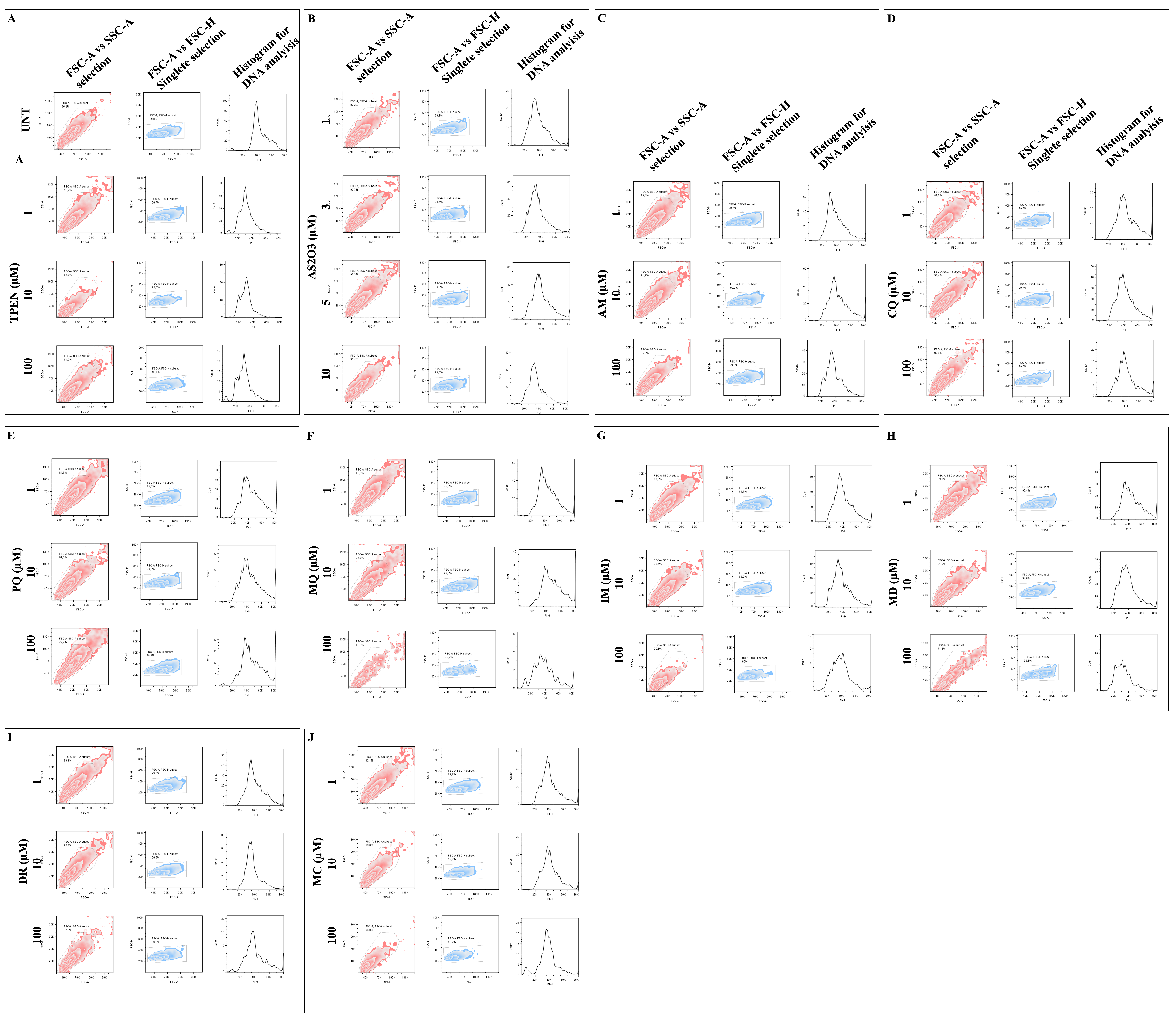
**

**Supplementary Figure 1. Gating strategy for cell cycle analysis.** Cells were incubated under the indicated experimental conditions, and flow cytometry analyses were performed as described in the Materials and Methods section. Representative density plots illustrate FSC-A/SSC-A gating for debris exclusion (red) and singlet discrimination (blue). Propidium iodide (PI) histograms (gray line) were used for cell cycle distribution analysis. Data are shown for untreated or TPEN-treated controls (A), and for cells treated with increasing concentrations of As₂O₃ (B), artemisinin (C), chloroquine (D), primaquine (E), mefloquine (F), ivermectin (G), moxidectin (H), doxorubicin (I), or minocycline (MC). Results are representative of three independent experiments.

**
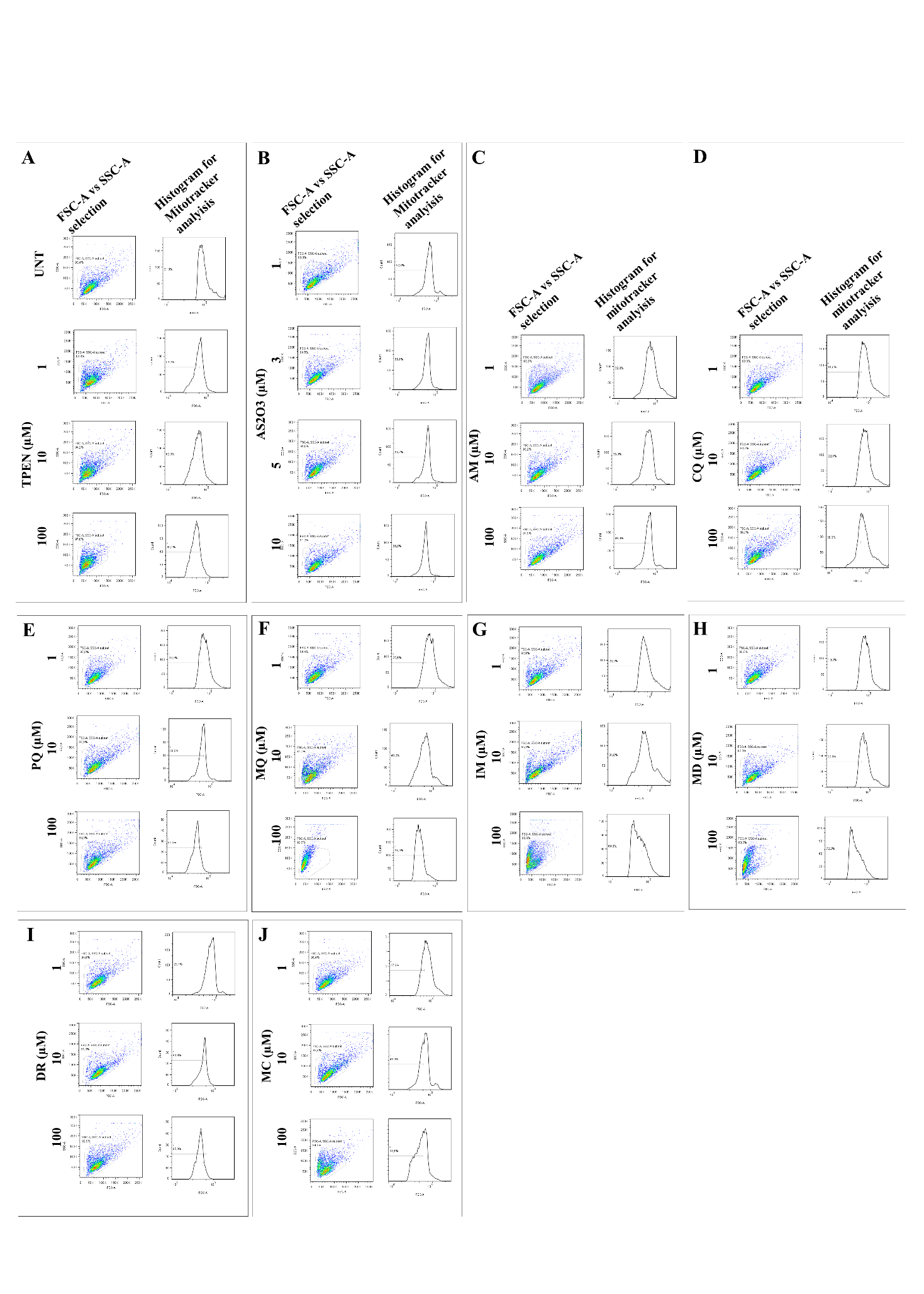
**

**Supplementary Figure 2. Gating strategy for Mitotracker analysis.** Cells were incubated under the indicated experimental conditions, and flow cytometry analyses were performed as described in the Materials and Methods section. Representative density plots illustrate FSC-A/SSC-A gating for debris exclusion (density plot). Mitotracker (MT) histograms (gray line) were used for mitochondrial membrane potential analysis. Data are shown for untreated or TPEN-treated controls (A), and for cells treated with increasing concentrations of As₂O₃ (B), artemisinin (C), chloroquine (D), primaquine (E), mefloquine (F), ivermectin (G), moxidectin (H), doxorubicin (I), or minocycline (MC). Results are representative of three independent experiments.
